# Unlocking the Potential of *Bacillus* Strains for a Two-Front Attack on Wireworms and Fungal Pathogens in Oat

**DOI:** 10.3390/insects17010028

**Published:** 2025-12-24

**Authors:** Aneta Buntić, Marina Dervišević Milenković, Jelena Pavlović, Uroš Buzurović, Jelena Maksimović, Marina Jovković, Magdalena Knežević

**Affiliations:** 1 Institute of Soil Science, Teodora Drajzera 7, 11000 Belgrade, Serbia; anetabuntic@gmail.com (A.B.); soils.pavlovic@gmail.com (J.P.); soilsbuzurovic@gmail.com (U.B.); jelena.maks@yahoo.com (J.M.); soils.jovkovic@gmail.com (M.J.); knez.magdalena@gmail.com (M.K.); 2 Institute of Pesticides and Environmental Protection, Banatska 31b, 11080 Belgrade, Serbia

**Keywords:** biocontrol, *Fusarium poae*, *Agriotes lineatus*, *Bacillus velezensis*, *Bacillus thuringiensis*, cereals, bio-inoculants, *Avena sativa* L.

## Abstract

(1) Background: Oat (*Avena sativa* L.) is a crop that is widely used in human nutrition, while it also plays an important role in animal husbandry as a high-quality forage crop. However, this crop is particularly susceptible to combined biotic stressors, including insect pests (*Agriotes lineatus*) and fungal infections (*Fusarium* spp.). These stresses act synergistically: root damage caused by wireworms increases the plant’s susceptibility to fungal infection, while pathogens further limit nutrient uptake and root system development. In recent years, the reduced efficacy of chemical pesticides against both insect pests and fungal pathogens has highlighted the need for alternative strategies in oat protection, leading to an increased focus on developing bacterial bio-inoculants as sustainable and effective biocontrol agents. (2) Methods: This study aimed to identify bacterial strains capable of suppressing wireworms (*Agriotes lineatus*) and *Fusarium* spp. in oats, while simultaneously promoting plant growth. Bacterial isolates were screened for key Plant Growth Promoting (PGP) and biocontrol traits, including IAA and siderophore production, phosphate solubilization, and the presence of toxin- and antibiotic-coding genes. (3) Results: The highest insecticidal effect against wireworms was recorded for *Bacillus velezensis* BHC 3.1 (63.33%), while this isolate also suppressed the growth of *F. proliferatum* for 59%, *F. oxysporum* for 65%, *F. poae* for 71%, and *F. graminearum* for 15%. The most effective *Bacillus* strains (with insecticidal and antifungal activity) were identified and tested in two pot experiments, where their ability to enhance plant growth in the presence of insects and fungi was evaluated under semi-controlled conditions. An increase in plant biomass, grain yield, and nitrogen content was observed in oat inoculated with *B. velezensis* BHC 3.1 and *B. thuringiensis* BHC 2.4. (4) Conclusions: These results demonstrate the strong potential of both strains as multifunctional bio-inoculants for enhancing oat growth and mitigating the adverse effects of wireworm damage and *Fusarium* infection.

## Simple Summary

Oats are an important crop for human nutrition and animal feed, but they can be damaged by both insects and fungi. In particular, wireworms and *Fusarium* fungi can attack the roots and other parts of the plant, making it weaker and reducing yield. Traditional chemical pesticides are becoming less effective against these pests and fungi, so scientists are looking for alternative, environmentally friendly solutions. In this study, we tested bacteria from the soil around oat roots to see if they could help plants grow better while also protecting them from pests and diseases. Two bacteria, *Bacillus velezensis* BHC 3.1 and *Bacillus thuringiensis* BHC 2.4, were especially effective. They reduced damage caused by wireworms and slowed the growth of several *Fusarium* fungi. When oats were treated with these bacteria in pot experiments, plants grew bigger, produced more seeds, and had higher nitrogen content than untreated plants. These findings show that these bacterial strains could be used as natural bio-inoculants to improve oat growth and protect against pests and diseases, offering a sustainable alternative to chemical pesticides.

## 1. Introduction

Oat (*Avena sativa* L.) is a cereal crop belonging to the Poaceae family, native to Western Asia [1]. Historically, this species has been cultivated on agricultural land for animal feed and prevalently for human consumption. Oat has high nutritional value, being rich in carbohydrates, vitamins (A, D, E, and B12), minerals (zinc, iron, calcium), protein, and particularly dietary fiber [2,3], among which beta-glucan is especially noteworthy, and has a low glycemic index. Oat also plays an important role in animal husbandry as a high-quality forage crop. In 2023, world production reached about 19 million tons, with Russia, Canada Poland, Finland and Australia as the leading producers (FAOSTAT, https://www.fao.org/faostat/en/#data/QCL/visualize (accessed on 30 October 2025)). In Serbia, about 40,000 tons of oats are produced annually, with an average grain yield of approximately 3000 kg/ha, which is above the world and European average yields [4].

Oats are often exposed to various insect pests, among which wireworms are the most important ones [5]. On the other hand, different types of fungi that produce mycotoxins [6,7], which can negatively affect plant growth and yield, as well as human health, pose a significant threat in oat production [6].

Wireworms (Coleoptera: Elateridae) cover a diverse group of insects, with almost 10,000 species described worldwide. In agricultural production, the species from the genus *Agriotes* are considered as the main pests [8]. Adult beetles usually only eat plants and rarely harm crops. However, the larval stages, wireworms, cause the most damage. They live in the soil and feed on the underground parts of plants. Wireworms damage a wide range of crops, including cereals [9], legumes [10], and vegetables [11]. Major global food crops like wheat, corn, and potatoes have suffered significant losses due to them [12,13,14]. Their feeding leads to thinner plant stands or poor-quality produce, especially in root and tuber vegetables [15,16].

Fungal infections caused by *Fusarium* species pose a significant threat to both oat production and consumption safety. Various *Fusarium* species produce mycotoxins, chemically stable secondary metabolites that can diminish the nutritional and commercial value of cereal grains [6]. Among these, deoxynivalenol, zearalenone, and fumonisin B1 are considered three of the five most important mycotoxins. Consequently, the European Union has established maximum allowable levels for several *Fusarium* toxins (European Commission. Commission Regulation No 1881/2006 setting maximum levels for certain contaminants in foodstuffs. OJEU 2006, 364, 5–24.). Fusarium head blight (FHB), as one of the most common fungal diseases, is associated with various plant-pathogenic *Fusarium* species, such as *F. graminearum*, *F. culmorum*, and *F. avenaceum*. Infection of oats with *F. graminearum* leads to loss of grain weight [17], and also reduces seed germination [18]. The pathogenic potential of *F. poae* is considered lower than that of *F. graminearum* [19]. However, Schöneberg et al. [20] reported that, between 2013 and 2015, a survey of commercially cultivated oats in Switzerland identified *F. poae* as the predominant species, while T-2/HT-2 toxins were detected as the most abundant mycotoxins. The results showed that oats grown after cereal crops, under reduced tillage, and in autumn-sown varieties contained higher concentrations of T-2/HT-2 and nivalenol (NIV) compared with those from ploughed fields and spring-sown varieties. These findings highlight the need for improved cropping practices and management strategies to effectively mitigate the risk of *F. poae* infection in oat grain.

Chemical pesticides are traditionally used in oat production to protect plants from diseases and pests [21], improve plant resistance and grain quality, and increase yield [22]. The use of fungicides suppresses fungi, but over time, fungi can develop resistance to these fungicides [23,24]. For this reason, increasing investment is being made in the development of bioinoculants containing bacteria such as *Bacillus* spp., *Pseudomonas* spp., and others [25]. These bacteria enhance plant growth and protect plants from pathogens. Due to both their effectiveness and environmentally friendly nature, bioinoculants are a good alternative to chemical pesticides. *Bacillus strains* exert its insecticidal effects primarily through the production of crystal proteins, known as Cry toxins, which are toxic to specific insect species [26]. Among the most commonly studied Cry proteins, Cry11 and Cry1B have shown activity against various insect pests, and recent studies suggest potential efficacy of these toxins against wireworm larvae [2]. These proteins accumulate as crystalline inclusions during sporulation, although some Cry variants, such as Cry3Aa, can also be expressed during vegetative growth [27,28]. Most *cry* genes are transcriptionally activated during sporulation and lead to the accumulation of Cry proteins as crystalline inclusions; however, certain variants, such as *cry3Aa*, are regulated by promoters active during vegetative growth and can be expressed prior to sporulation [29]. When an insect ingests *B. thuringiensis* spores containing these crystal proteins, the crystals are solubilized in the alkaline environment of the insect midgut. The protoxins are then proteolytically cleaved by midgut enzymes, such as trypsin- and chymotrypsin-like proteases, to generate active toxins [30]. The activated toxins bind to specific receptors on the midgut epithelial cells, including cadherins, aminopeptidases, and alkaline phosphatases, undergoing conformational changes that enable insertion into the apical membrane. This insertion results in the formation of pores or ion channels, leading to leakage of ions and nutrients, cell swelling, and lysis [31]. In addition, the interaction with receptors and pore formation disrupts the brush border structure, impairs digestion, and can cause gut paralysis, preventing further feeding. The combined effects of nutrient loss, tissue damage, dehydration, and systemic infection ultimately lead to insect death [32,33,34]. Besides Cry11 and Cry1B, other Cry toxins could be important for controlling coleopteran pests, including various beetle larvae, highlighting their relevance in targeting insects of this order. In addition, our previous research demonstrated that different *Bacillus* strains, including *Bacillus velezensis*, had both antifungal and insecticidal effect against *F. poae* and wireworms in barley and wheat, respectively [35,36].

Therefore, this research was conducted with the aim of finding appropriate bacterial strains that could be used for the suppression of wireworms and *Fusarium* species in oat, while simultaneously promoting its growth. The isolation of bacteria from rhizosphere soil was performed, followed by the evaluation of bacterial traits with potential in plant growth promotion (production of IAA, siderophores and phosphate solubilisation activity) and in the biocontrol of *Agriotes lineatus* larvae (presence of toxin-coding genes and bioassays under laboratory conditions) and *Fusarium* species (presence of AMPs genes and in vitro antifungal assay). In addition, effective strains were identified to the species level and applied as bio-inoculants in two parallel pot experiments (pot experiment 1 and pot experiment 2), where their PGP and biocontrol effects were evaluated under semi-controlled conditions.

## 2. Materials and Methods

### 2.1. Bacterial Strains

In this research, isolation of bacteria was performed from rhizosphere soil collected on five cereal production fields in the Republic of Serbia during the spring of 2024. Isolation of bacteria was performed on nutrient agar (NA), by inoculation of 500 µL of soil decimal solution (10^−5^), followed by 24 h of incubation on 28 °C. All bacterial isolates were re-streaked on NA until the pure culture of each isolate was obtained, and isolates were stored on inclined NA on 4 °C. In addition to the newly isolated bacteria, strain *B. thuringiensis* BHC 2.4 was also included in this research due to previously demonstrated insecticidal activity against wireworms [35]. For all further tests, working cultures of bacterial isolates were grown on NA for 24 h on 28 °C or in nutrient broth (NB) on 28 °C on rotary shaker (150 rpm) until the concentration of 10^9^ CFU mL^−1^ was achieved.

### 2.2. Plant Growth Promoting Traits of Bacteria and AMPs Gene Detection

To screen bacterial isolates for PGP traits, the production of indole-3-acetic acid (IAA), and siderophores, as well as the ability of bacterial isolates to solubilize phosphates, were evaluated. The production of IAA was evaluated by using Salkowski reagent in Nutrient broth with (2 mg mL^−1^) and without tryptophan enrichment by following a protocol described by Gordon and Weber [37]. In addition, the ability of isolates to produce siderophores was evaluated on Chrome Azurol (CAS) agar based on the [38], while the ability of isolates to solubilize inorganic phosphates was determined on Pikoskaya agar by following a procedure described in Rokhbakhsh-Zamin et al. [39]. All tests were performed in triplicates, and the results were expressed as mean values ± standard deviation.

Further steps included detection of the presence of bacterial genes with potential role in biocontrol activity against phytopathogenic fungi by PCR method. Namely, the presence of genes coding for antimicrobial peptides (AMPs)-lipopeptides such as *srfAA* (surfactin), *bacA* (bacylisin), *fenD* (fengycin), *bmyB* (bacyllomicin), *spaS* (subtilin) and *ituC* (iturin) was evaluated. Isolation of the total genomic DNA for all molecular methods was performed by using CTAB protocol described in Dimkić et al. [40]. The amplification of AMP genes was performed based on the protocols described by Mora et al. [41]. The presence of bands for each tested gene was checked on the specific position on the electrophoresis gel in comparison to the DNA ladder (Thermo Scientific (Waltham, MA, USA) GeneRuler 1 kb DNA Ladder). The presence of band on the specific position (Table S1) was considered as a positive result.

### 2.3. Insecticidal Potential

For the determination of insect activity of bacterial isolates, larvae of *Agriotes lineatus* were collected from the fields with their natural occurrence. Wireworm larvae were collected from the localities of Smederevska Palanka (42°21′17″ N, 20°56′55″ E) and Despotovo (45°46′73″ N, 19°57′20″ E), in places where an increased number of individuals had been observed in previous years. From there, they were brought to the laboratory and reared until the moment of use in the previously described manner [35].

Bacterial isolates were screened for the presence of genes coding for proteins with insecticidal effect, by PCR method and based on the visualization of bands of expected size (Table S1) on the specific position on electrophoresis gel. The selection of *cry11*, *cry1B*, *vpb*, *vpa*, and *cyt* genes was based on their general insecticidal activity, which may occasionally show cross-order effects, providing preliminary insight into the insecticidal potential of the strains [42]. Additionally, *vpb* and *vpa* have been shown to act together to exert toxicity against coleopteran pests such as *Diabrotica* spp., and certain Cyt proteins can also display coleopteran activity and synergize the effects of Cry toxins [43]. Screening for this broader set of genes provided preliminary insight into the pesticidal potential of isolates against wireworms, particularly when specific coleopteran toxins are not yet fully characterized.

The PCR amplification of genes coding for crystal proteins (*cry11* and *cry1B*) was performed based on Jain et al. [44] and Thammasittirong and Attathom [45]; amplification of gene for vegetative insecticidal protein gene (*vpb* and *vpa*) based on Senthilkumar et al. [46], and amplification of cytolytic toxin gene based on Ibarra et al. [47].

The insecticidal activity of the tested strains was evaluated under laboratory conditions, simulating field conditions. For the evaluation of insecticidal effect of bacteria, bacterial cultures were prepared by growing isolates for 96 h on 28 °C in order to provide optimal time frame for the development of insecticidal proteins. Oat seeds were soaked in bacterial cultures for 5 min, then air-dried and left in Petri dishes to germinate for 72 h. In the control treatment, oat seeds were soaked in sterile distilled water. After 72 h, about 200 g of soil were added to each Petri dish to imitate natural conditions for wireworms. Then, 50 oat seedlings and 10 adult *A. lineatus* larvae of the same age (body length 15–20 mm), which had been starved for 48 h, were placed in each dish. Each treatment was performed in five replicates. The Petri dishes were left in a dark place, and the mortality of the individuals was determined daily for the next 10 days.

After the experiment was ended, the isolation of bacteria from the cadavers of *A. lineatus* larvae was performed in order to confirm the presence of bacterial isolates in bacterial treatments which have shown insecticidal activity. The isolation of bacteria was performed by following a modified protocol described by Danismazoglu et al. [48]. The diseased larvae from each treatment were surface sterilized by emerging in 70% ethanol for 2 min, rinsed two times in sterilized distilled water and homogenized by a glass rod. Serial dilutions of homogenized material were prepared in sterile sodium chlorine (NaCl), and 500 µL of 10^−5^ dilution was inoculated on NA. After the incubation period (48 h on 28 °C), the grown isolates were characterized based on the Gram stain and colony morphology.

### 2.4. Antifungal Potential

For the determination of biocontrol potential of bacterial strains, the ability of bacteria to suppress the growth of four phytopathogenic *Fusarium* spices (*F. poae*, *F. graminearum*, *F. proliferatum* and *F. oxysporyum*) was evaluated in vitro, by dual culture method on Potato dextrose agar (PDA). Fungal working cultures were prepared on PDA medium (72 h on 28 °C). A plug of freshly grown fungal mycelia (5 mm diameter) was placed in the center of the Petri dish, and 20 µL of bacterial suspensions (10^9^ CFU mL^−1^) were near the edges of the Petri dish in quadruplicates. For the control sample, no bacterial suspension was applied. The plates were then incubated for 72 h on 28 °C, after which the inhibition of fungal growth was measured both on the control and bacterial suspensions plates in mm. For each isolate, three independent replications were performed, and the results were expressed as mean values of the percentage of inhibition of mycelial growth ± standard deviation, based on the following formula: Inhibition% = (Fungal control–Bacterial treatment)/Fungal control × 100% [49].

### 2.5. Molecular Identification of Potentially Effective Isolates

For the isolates which have shown at least one significant trait (as described in Section 2.2, Section 2.3 and Section 2.4, molecular identification to the species level was performed. The first step included the amplification of 16S rRNA sequence by using P_0_ and P_6_ DNA primer pair (Metabion, Planegg, Bavaria, Germany), based on the previously described protocol [50]. Subsequent step of molecular identification to the species level included amplification of house-keeping *tuf* gene by using tufGPF and tufGPR DNA primers, as described by Draganić et al. [51]. All DNA amplification were performed by using PCR Master Mix (Qiagen, Hilden, Germany) and in Eppendorf™ Mastercycler X50s 96-Well Silver Block Thermal Cycler (Eppendorf, Hamburg, Germany). Purification of obtained PCR products, as well as the DNA sequencing, was performed by using commercial service (ECO-seq) of Macrogen Europe, The Netherlands. The obtained DNA sequences were then checked for quality and deposited in NCBI (National Center for Biotechnology Information) database in order to obtain accession numbers. Based on the obtained sequences and by comparation to the sequences available in NCBI, the identification of isolates to the species level was performed.

### 2.6. Pot Experiments

For the confirmation of PGP ability of the most efficient bacterial strains, as well as their biocontrol potential, two pot experiments were set up: one for the biocontrol activity against *Agrotes lineatus* larvae (pot experiment 1) and one for PGP and biocontrol activity against *F. poae* (pot experiment 2). Bacterial strain BHC 2.4 was selected based on previously demonstrated insecticidal activity, while the isolate BHC 3.1 was selected based on its efficiency against *F. poae* showed in in vitro experiments. Both experiments were set up at the same time (experiment start date: 29 May 2025; experiment end date: 1 August 2025) with the same amount of growing substrate which was placed in pots (15 cm diameter). The growing substrate with the properties presented in Table S2 was used for both pot experiments.

For experiment 1, pest application (P), bacterial inoculation and pest application (B+P) and control (C) treatments were applied. Each treatment was performed in three replicates. Oat seeds were soaked in bacterial cultures (10^9^ CFU mL^−1^) for 10 min, air-dried and then placed in Petri dishes for germination for 48 h, the same time that the *A. lineatus* larvae were starved. After two days, 15 seedlings per treatment and 10 wireworm larvae were placed in pots filled with soil. The pot experiment 1 was set up in a randomized system, in the light chamber (25 °C and 65% relative humidity) at the Institute of Pesticides and Environmental Protection, Belgrade. At the end of pot experiment 1, the number of plants was calculated, and the total fresh and dry plant biomass (g) was measured, including the mass of seeds in the spikes.

For the pot experiment 2, bacterial inoculation (B), fungal infection (F), bacterial inoculation and fungal infection (B + F) and control (C) treatments were applied. All treatments were set-up in triplicates. The amount of 15 oat seeds was used for each pot. Seeds were inoculated by bacterial strains, by shaking in bacterial culture (10^9^ CFU mL^−1^) for 15 min, and air drying. Infection of seeds by *F. poae* was performed by using fungal spore suspension, while for the control treatment no infection and inoculation were applied. The seeds for each treatment were then placed in pots filled with soil and covered with a thin layer of substrate. The pot experiment 2 was set up in a randomized system, in the greenhouse at the Institute of Soil Science, Belgrade. At the end of the pot experiment 2 the content of chlorophyll (Chl: arbitrary units reflecting relative chlorophyll content in the leaf), as well as the indexes of anthocyanins (Anth: arbitrary units, indicating relative accumulation of anthocyanin pigments), flavonols (Flav: arbitrary units, indicating relative accumulation of flavonoid compounds) and nitrogen balance index (NBI: index representing the balance between leaf chlorophyll and flavonoid content) in plants were measured by Dualex Scientific leafclip sensor for each plant with surface larger than 20 mm^3^. The differences between treatments were evaluated (B, F, B + F and C) for each measured parameter (NBI, Chl, Flav and Anth). These parameters are expressed as unitless index values, as the Dualex instrument provides relative optical indices rather than absolute pigment concentrations. Further, the overall fresh and dry plant biomass was measured (g), including the mass of seeds in spikes. After the harvest, several spikes (surface sterilized by washing in 70% ethanol and rinsing in sterile distilled water) from each treatment were placed on the Petri dish with PDA medium to detect the *F. poae* infection. Plates were incubated for 72 h at 28 °C, and grown fungi were evaluated based on the colony morphology.

For both experiments, the collected plant material was dried in a drying oven at 65 °C, and grinded by analytical mill. The content of nitrogen (N%) in shoots and spikes was determined using an elemental CNS analyzer, Vario model EL III (Elementar Analysensysteme GmbH, Langenselbold, Germany) [52].

### 2.7. Statistical Analysis

For all analyses, one-way ANOVA was used, followed by Duncan’s multiple range test as a post hoc analysis to determine significant differences between treatments. Data are presented as mean ± standard deviation (SD) for both insecticidal activity and the parameters from the pot experiments (yield and nitrogen content). 

## 3. Results

### 3.1. Plant Growth Promoting and Biocontrol Potential of Bacterial Isolates

**Figure 1:**
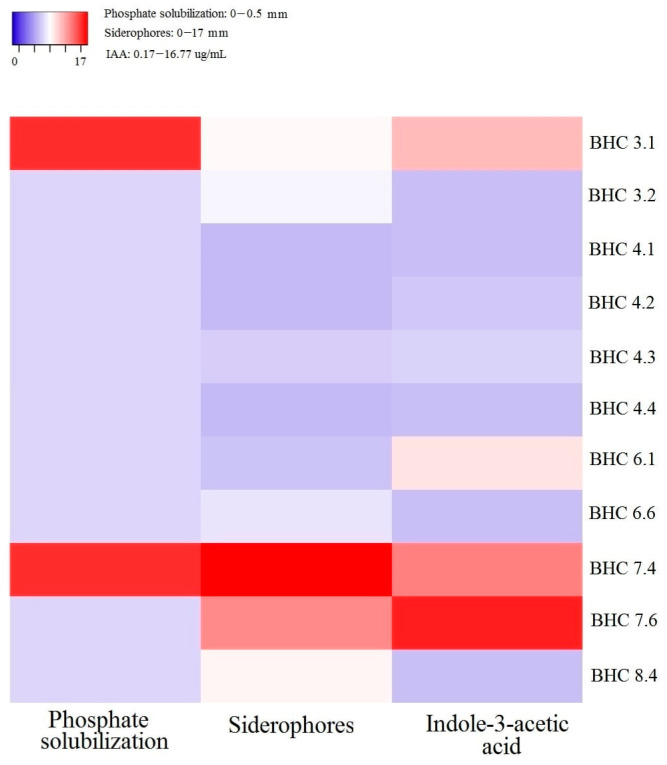
Plant growth promoting (PGP) traits of bacterial isolates.

The total of 71 bacterial isolates was isolated from 5 soil samples, which were used as the working bacterial collection for further experiments. Out of all isolated bacteria, only 11 isolates showed at least one PGP trait (Figure 1). This was considered as the initial screening step in finding multifunctional bacterial inoculant (with PGP and biocontrol potential), and these isolates were used for further phases of the experiment.

The results of PGP bacterial traits showed that the values for IAA production ranged from 0.17 (BHC 4.4) to 16.77 µg mL^−1^ (BHC 7.6), for siderophores from 0 (BHC 4.1; BHC4.2; BHC 4.4) to 17 mm (BHC 7.4), while for phosphate solubilization only isolates BHC 3.1 and BHC 7.4 showed halo zone of 0.5 mm. In addition, only two isolates (BHC 3.1 and BHC 7.4) had all three tested PGP traits (Figure 1).

**Figure 2:**
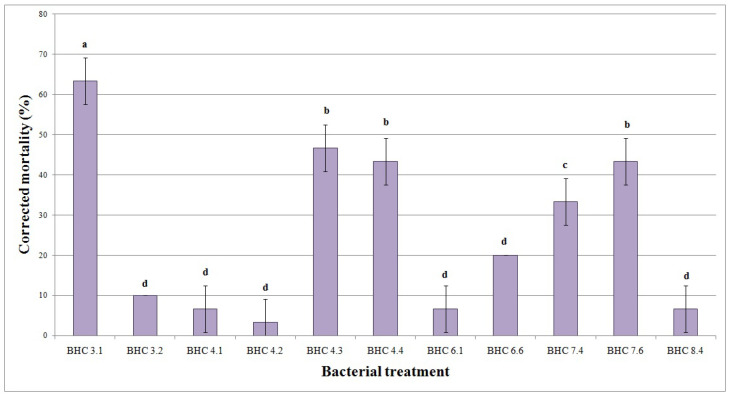
Mortality (%) of *Agriotes lineatus* larvae induced by different bacterial treatments. a–d: Means followed by the same superscript letters are not significantly different according to Duncan’s multiple range test (*p* ≤ 0.01). Bars above means represent standard deviation.

In bioassays, the activity of eleven bacterial strains against *A. lineatus* larvae was individually evaluated. All tested strains exhibited higher mortality rates than the control group. Based on the mortality data 10 days after treatment, it can be concluded that the highest and statistically significantly different efficacy of 63.33% was recorded in the treatment with the BHC 3.1 strain. It was followed by strains BHC 4.3, BHC 4.4, and BHC 7.6 with an efficiency from 43.33% to 46.67%. The strain BHC 7.4 showed an efficacy of 33.33%, and all other strains (BHC 3.2, BHC4.1, BHC 4.2, BHC 6.1, BHC 6.6, BHC 8.4) exhibited an efficacy between 6.67% and 13.33% (Figure 2).

**Figure 3:**
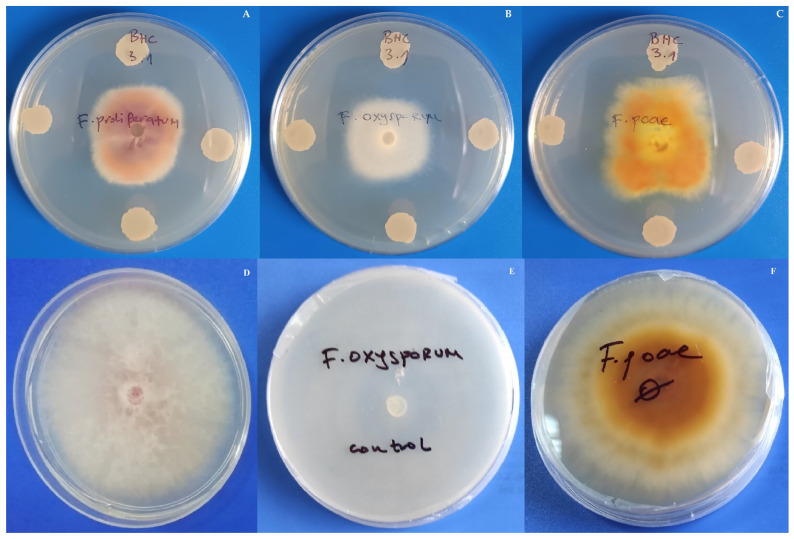
Antagonist effect of isolate BHC 3.1 against *F. proliferatum* (**A**), *F. oxysporum* (**B**) and *F. poae* (**C**)—upper row< and *F. proliferatum* (**D**), *F. oxysporum* (**E**) and *F. poae* (**F**) fungal controls—lower row.

Regarding the antifungal activity of bacterial isolates, only isolate BHC 3.1 showed antagonistic effect against all four tested fungi. Namely, this isolate suppressed the growth of *F. proliferatum* for 59%, *F. oxysporum* for 65%, *F. poae* for 71%, and *F. graminearum* for 15% (Figure 3). In addition, only isolates BHC 4.1 and BHC 4.3 showed antagonistic effect against *F. oxysporum* and suppressed its growth for 55% and 47%, respectively. Other bacterial isolates had no activity against tested fungi.

Out of all tested bacterial isolates, only for the isolate BHC 3.1 the presence of genes coding for bacyllomicin, fengycin, surfactin and subtilin was confirmed. On the other hand, the presence of genes coding for insecticidal proteins was not detected in the tested bacterial isolates.

### 3.2. Molecular Identification of Bacterial Isolates

By comparing the obtained DNA sequences of 16S rRNA and housekeeping (*tuf*) genes of bacterial isolates to the ones available in the NCBI base, bacterial isolates were identified as *B. velezensis*, *B. mycoides*, *B. pumilus*, *B. pseudomycoides*, *B. safensis*, *Peribacillus frigotolerans*, *B. pseudomycoides*, *B. toyonesis*, *B. thuringinesis* (Table 1).

### 3.3. Impact of Bacillus Inoculation on Oat Plants Infested by Agriotes lineatus

**Figure 4:**
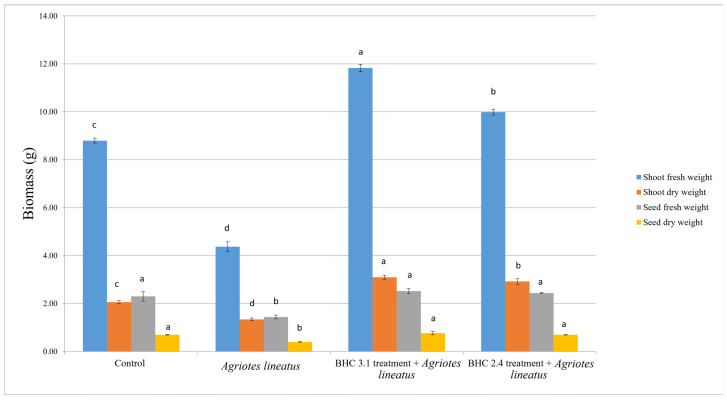
Impact of bacterial treatments (*B. velezensis* BHC 3.1 and *B. thuringiensis* BHC 2.4) and insect infection (*Agriotes lineatus* larvae)on the yield parameters of oat plants. Means followed by the same superscript letters are not significantly different according to Duncan’s multiple range test (*p* ≤ 0.01). Bars above means represent standard deviation.

The results of pot experiments showed that the values for all measured yield parameters were the lowest for the plants where only *A. lineatus* larvae were applied (Figure 4).

The average yield values for fresh and dry matter of oats were statistically significantly different across all treatments. The highest values for fresh matter (11.83 ± 0.15 g) and dry matter (3.1 ± 0.08 g) were recorded in the *B. velezensis* BHC 3.1 treatment. This was followed by the *B. thuringiensis* BHC 2.4 treatment, where the fresh matter yield was 9.99 ± 0.12 g and the dry matter yield was 2.92 ± 0.12 g. The lowest yield values were recorded in the treatment where only *Agriotes linaetus* larvae were applied, where the fresh matter yield was 4.36 ± 0.21 g and the dry matter yield was 1.34 ± 0.06 g.

The highest values for fresh and dry mass of oat seeds were recorded in the *B. velezensis* BHC 3.1 treatment, followed by the *B. thuringiensis* BHC 2.4 treatment, while the lowest values were again recorded in the treatment where only *Agriotes lineatus larvae* were applied.

### 3.4. Impact of Bacillus Inoculation on Oat Plants Infected by Fusarium poae

**Figure 5:**
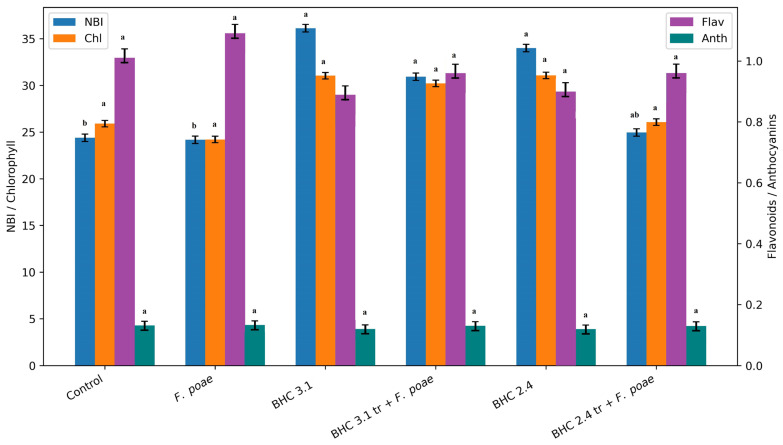
Impact of bacterial treatments (*B. velezensis* BHC 3.1 and *B. thuringiensis* BHC 2.4) and fungal infection (*F. poae*) on the physiological parameters of oat plants. NBI, Chl, Flav and Anth expressed as unitless index values, as the Dualex instrument provides relative optical indices rather than absolute pigment concentrations. Flav and Anth were plotted at 10-fold magnification to ensure better visibility. Means followed by the same superscript letters are not significantly different between treatments for each measured variable (NBI, Chl, Flav and Anth) according to Duncan’s multiple range test (*p* ≤ 0.01). Bars above means represent standard deviation.

The results of plant-physiological parameters showed that the highest NBI content was recorded for treatments with or without fungal infection, while the lowest values were recorded for the control and *F. poae* infected plants (Figure 5). Values of chlorophyll content were not statistically different between treatments. On the other hand, flavonoid and anthocyanin content were not statistically different between treatments. However, the highest values were detected in *F. poae* plants, and the lowest in bacterial treatments, while the treatment of infected plants decreased Flav and Anth content in compassion to the infected plants.

**Figure 6:**
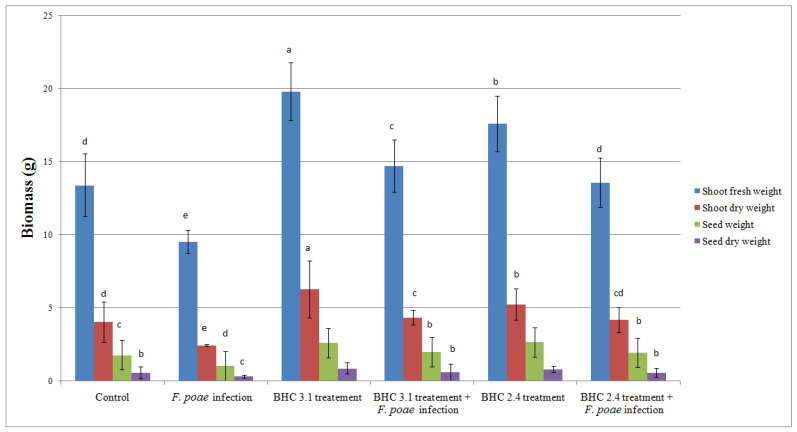
Impact of bacterial treatments (*B. velezensis* BHC 3.1 and *B. thuringiensis* BHC 2.4) and fungal infection (*F. poae*) on the yield parameters of oat plants. Means followed by the same superscript letters are not significantly different according to Duncan’s multiple range test (*p* ≤ 0.01). Bars above means represent standard deviation.

The shoot fresh and dry weight of oat, as well as the yield of spike seeds were affected both by bacterial inoculation (*B. velezensis* BHC 3.1 and *B. thuringiensis* BHC 2.4) and fungal infection (*F. poae*) (Figure 6). For the non-infected plants, the shoot dry weight in treatments by both *B. velezensis* BHC 3.1 and *B. thuringiensis* BHC 2.4 was significantly increased in comparison to the control. Similar results were recorded for seed shoot dry weight where the bacterial treatments induced the increase in both parameters in comparison to the control. For the infected plants, the lowest yield of fresh and dry shoot weight and spike seeds was detected for the *F. poae* infection, while the treatment of oat by bacterial strains induced an increase in yield of both infected and uninfected plants.

**Figure 7:**
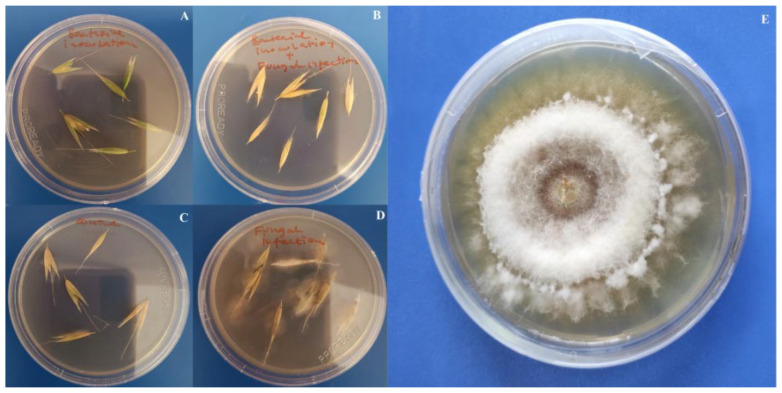
Emergence of *F. poae* on spikes of infected plants and pure culture on PDA medium: (**A**)—bacterial treatment, (**B**)—bacterial treatment and fungal infection, (**C**)—control, (**D**)—fungal infection, (**E**)—pure culture of *F. poae* grown from fungal infection treatment spikes (PDA).

The emergence of *F. poae* was detected on PDA medium after 24 h from the surface sterilized spikes of oat (Figure 7D), while no fungal growth was detected on medium where the spikes from control (Figure 7C), bacterial treatment (Figure 7A) and bacterial treatment and fungal infection (Figure 7B) were inoculated.

Regarding the nitrogen content in the grains of oat from the experiment 1, it was observed that the lowest values were recorded for the *Agriotes lineatus* treatment (0.86%), as presented in Table S3. The highest values of N% were recorded in the grains of oat infected with *Agriotes lineatus* and treated with *B. thuringiensis* BHC 2.4 (1.46%). Similar results were recorded in the pot experiment 2. The seeds infected with *F. poae* had the lowest values of N% (1.77%), while the bacterial treatment of infected seeds both by *B. velezensis* BHC 3.1 and *B. thuringiensis* BHC 2.4 increased N% (1.9% and 2.01%, respectively). In addition, the treatment of non-infected oat seeds by *B. velezensis* BHC 3.1 and *B. thuringiensis* BHC 2.4 increased N% in comparison to the non-infected control to 2.1% and 2.16%, respectively.

## 4. Discussion

Oats are particularly susceptible to combined biotic stressors, such as *Agriotes lineatus* larvae and *F. poae* infection [5,6]. These stresses act synergistically: root damage caused by wireworms increases the plant’s susceptibility to fungal infection, while pathogens further limit nutrient uptake and root system development. Therefore, this research aimed to find an eco-friendly alternative to chemical interventions that could be used for the suppression of wireworms and *Fusarium* species in oat, while simultaneously promoting its growth.

In general, the insecticidal activity of *Bacillus* spp. is particularly effective against larvae of Lepidoptera, Diptera, and Coleoptera; however, different strains of *B. thuringiensis* exhibit varying degrees of specificity and efficacy depending on the insect species targeted [53]. In our investigation, we evaluated several *Bacillus* strains for their insecticidal activity against wireworms, a notorious pest in agricultural systems. The strain *Bacillus velezensis* BHC 3.1 demonstrated an insecticidal efficiency of 63.33%, while other tested strains showed efficiencies ranging from 6.67% to 46.67%. Previous studies also highlight the efficacy of *Bacillus* strains against these insect pests. In our previous research, *B. velezensis* BHC 5.6 demonstrated significant insecticidal activity against *Agriotes lineatus* larvae in barley, with a mortality rate of 56.67%, indicating its potential as an effective biocontrol agent [36].

Of particular note, *B. thuringiensis* strain BHC 2.4, which carries the *cry11*, *cyt2*, and *krsA* genes, exhibited significant insecticidal activity. This strain was also able to produce siderophores, protease, amylase, and cellulase, which may further enhance its insecticidal potential. In terms of mortality, inoculations with the *B. thuringiensis* strains resulted in mortality rates from 6.67% to 72.22% in wheat [35]. Future research on evaluating bacterial properties significant in biocontrol of wireworms should also include screening of the presence of Cry3, Cry7, Cry8 and binary Cry34/35 genes [42].

In the present study, *Bacillus velezensis* BHC 3.1, which showed the highest insecticidal effect (63.33%), was negative for the presence of toxin genes. On the other hand, it was positive for the presence of genes coding for bacyllomicin, fengycin, surfactin and subtilin. Although the production of *Bacillus* lipopeptides was previously tightly related to their antifungal activity, these findings indicate that they could also be responsible for the insecticidal effect of antibiotic-producing strains. Recently, it has been demonstrated that *B. velezensis* isolates can exhibit insecticidal activity even in the absence of classical Cry toxin genes. This activity is attributed to the production of secondary metabolites, including lipopeptides such as surfactin, iturin, and fengycin, which can disrupt insect cell membranes and interfere with physiological processes [54]. These compounds may induce enzymatic disturbances, feeding inhibition, and tissue damage, ultimately leading to insect mortality, and often act synergistically to enhance the overall effect [55]. Such mechanisms make *B. velezensis* a promising candidate for the development of biological insecticides in agriculture. Antagonistic effect of *B. velezensis* strains against different species of phytopathogenic fungi is most commonly explained by the production of antibiotics. Besides antibiotic production, presence of fungal cell-wall-degrading enzymes such as protease and cellulase, as well as PGP substances such as siderophores and IAA, could be responsible for the antifungal effect of *B. velezenisis* against wheat root rot in a greenhouse [56]. Similarly, Chen et al. [57] demonstrated antagonist effects of *B. velezenisis* (phosphate-solubilizing, siderophore-producing and with genes encoding for surfactin, fengycin, butirosin, macrolactin H, bacillibactin, difficidin, and bacilysin), showing suppressive effect on *F. oxysporum*, as well as on *Magnaporthe oryzae* and *Rhizoctonia solani*. Cheng et al. [58] found that *B. velezensis* originating from oats, with the ability to produce indole-3-acetic acid (IAA), form biofilms, and fix nitrogen can inhibit some of the common fungal diseases in oat such as powdery mildew, as well as *Bipolaris sorokiniana* and *Tilletia controversa.*

In the present study, *B. velezensis* BHC 3.1 was the only strain for which the presence of bacyllomicin, fengycin, surfactin and subtilin was detected. The production of these antibiotics could be responsible for its strong effect against tested *Fusarium* species. The biocontrol activity of other *B. velezensis* antibiotic-producing strains was also previously confirmed. Biocontrol potential of PGP *B. velezensis* strain against *F. poae* was recorded in a pot experiment, where the yield of barely infected by *F. poae* was increased by up to 10.12%, when bacterial inoculation was applied [36]. In addition, the presence of *fenD*, *bmyB*, *srfAA*, *spaS* genes was detected in this efficient strain. In a pot experiment conducted by Shan et al. [59], EPS, SID, IAA and ACC deaminase-producing *B. velezensis* reduced rice bacterial blight caused by *Xanthomonas oryzae* pv. *Oryzae* by up to 63.53%, by simultaneously promoting plant growth, and inducing systemic resistance in rice plants. Our results showed that inoculation of oat seeds infected with *F. poae* by *B. velezensis* BHC 3.1 increased shoot fresh weight up to nearly 55%, in comparison to the infected control. Similarly, the seed fresh wight of infected seeds was increased for up to 90% by *B. velezensis* BHC 3.1. These results indicate that the *B. velezensis* BHC 3.1 has the potential to significantly increase the yield of oat infected with *F. poae*. Similar pattern was also observed for the nitrogen content (N%) in oat plants (both infected with *F. poae* and *A. lineatus*), measured during the plant developmental phase (by Dulaex LeafClip) and at the end of the pot experiment. Other research suggested that the inoculation of plants by PGP bacteria from *Bacillus* genus (such as *B. subtilis*, *B. amyloliquefaciens* and *B. velezensis*) can improve root development which consequently improves nutrient uptake, including nitrogen [60]. In addition, *Bacillus* species are known to enhance nitrogen availability through organic matter mineralization and to stimulate plant enzymatic systems involved in nitrogen assimilation. Activation of induced systemic resistance (ISR) by *Bacillus*-derived lipopeptides may also contribute to reduced oxidative stress and protection of nitrogenous compounds. This is especially significant under unfavorable growth conditions, including fungal infections, where nutrient uptake and protein synthesis is often inhibited.

On the other hand, inoculation of oat plants infected with *A. lineatus* larvae using *B. velezensis* BHC 3.1 and *B. thuringiensis* BHC 2.4 resulted in higher nitrogen content compared with plants exposed to the insect without bacterial treatment. These results suggest that PGP *Bacillus* strains can mitigate the negative impact of root damage caused by wireworms through their bio-stimulatory and bio-protective effects. The observed increase in nitrogen may be related to improved root growth and nutrient uptake promoted by bacterial synthesis of phytohormones such as indole-3-acetic acid. Moreover, the entomopathogenic activity of *B. thuringiensis* could have limited the damage caused by *A. lineatus*, further preserving root functionality. Altogether, these findings indicate that inoculation with multifunctional *Bacillus* strains supports nitrogen balance and overall plant resilience under biotic stress, highlighting their potential use in integrated biocontrol and biofertilization strategies for cereal crops.

In both experiments, involving oat plants infected with *A. lineatus* larvae or *F. poae*, the nitrogen content in seeds was generally low. This is consistent with the fact that the trials were terminated during early developmental stages, before protein accumulation in the seeds occurred. At these stages, most nitrogen is allocated to vegetative growth and metabolite synthesis, while transport to the developing seed is minimal [61]. Despite the low baseline nitrogen, inoculation with *B. velezensis* BHC 3.1 and *B. thuringiensis* BHC 2.4 increased nitrogen content in both trials. This suggests that PGP bacteria can enhance nitrogen uptake and assimilation even at early growth stages, when plants have limited capacity for nutrient storage in seeds. The effect may be particularly pronounced under biotic stress, such as root damage from wireworms or infection by *F. poae*, highlighting the potential of early bacterial inoculation to improve nutrient status and support later protein accumulation in seeds.

## 5. Conclusions

The results of this study indicate that inoculation of oat plants with *B. velezensis* BHC 3.1 and *B. thuringiensis* BHC 2.4 significantly improves plant growth, grain yield, and seed mass under stress caused by *A. lineatus* larvae and *F. poae* infection. These strains exhibit a multifaceted effect by mitigating the negative impacts of pests and pathogens while maintaining the plant’s nutritional status, including nitrogen content. Based on these findings, both strains represent highly promising candidates for the development of bio-inoculants aimed at sustainable oat production under increased biotic pressure. Further field studies are essential to confirm the efficacy and stability of these strains under real agroecosystem conditions, including different oat cultivars and soil types, as well as interactions between pest, fungal pathogen and bacterial treatments. Optimization of application strategies, such as timing, dosage, and potential synergistic effects with other PGP microorganisms or agronomic practices, is recommended. In the long term, the use of these bio-inoculants could contribute to sustainable improvements in oat yield and seed quality, while reducing damage from pests and pathogens and minimizing reliance on chemical fertilizers and pesticides.

## Figures and Tables

**Table 1:** Identification of bacterial isolates based on the 16S rRNA and *tuf* gene sequence.

Bacterial Isolate	Identity 16S rRNA	Identity *tuf*	NCBI Accession Number	Species
BHC 3.1	99.89%	99.83%	PX482504	*B. velezensis*
BHC 3.2	99.53%	99.82%	PX482503	*B. mycoides*
BHC 4.1	100%	100%	PX482507	*B. pumilus*
BHC 4.2	100%	99.56%	PX482521	*B. pseudomycoides*
BHC 4.3	100%	100%	PX482522	*B. safensis*
BHC 4.4	100%	99.78%	PX482523	*Peribacillus frigotolerans*
BHC 6.1	100%	99.60%	PX482525	*B. pseudomycoides*
BHC 6.6	99.83%	100%	PX482528	*B. toyonensis*
BHC 7.4	99.60%	100%	PX482529	*B. toyonensis/B. thuringiensis*
BHC 7.6	100%	100%	PX482535	*B. mycoides*
BHC 8.4	99.88%	100%	PX482537	*B. pseudomycoides*

## Data Availability

Sequence data have been deposited in the NCBI under the following accession numbers: *B. velezensis* BHC 3.1-PX482504, *B. mycoides* BHC 3.2-PX482503, *B. pumilus* BHC 4.1-PX482507, *B. pseudomycoides* BHC 4.2-PX482521, *B. safensis* BHC 4.3-PX482522, *Peribacillus frigotolerans* BHC 4.4-PX482523, *B. pseudomycoides* BHC 6.1-PX482525, *B. toyonensis* BHC 6.6-PX482528, *B. toyonensis/B. thuringiensis* BHC 7.4-PX482529, *B. mycoides* BHC 7.6-PX482535, *B. pseudomycoides* BHC 8.4-PX482537.

## Supplementary Materials

The following supporting information can be downloaded at: https://www.mdpi.com/article/10.3390/insects17010028/s1, Table S1. DNA primers used for antibiotic and toxin gene presence screening and their expected product size (bp). Table S2. Chemical analysis of the growing substrate used for pot experiments 1 and 2. Table S3: Impact of *B. velezensis* BHC 3.1 and *B. thuringiensis* BHC 2.4 on nitrogen percentage in oat seeds and shoots.

## Abbreviations

The following abbreviations are used in this manuscript:

PGP	plant growth-promoting
PGPR	plant growth-promoting Rhizobacteria
NA	nutrient agar
EPS	exopolysaccharides
SID	siderophores
IAA	indole-3-acetic acid
CAS	Chrome Azurol agar
Flav	arbitrary units, indicating relative accumulation of flavonoid compounds
Anth	arbitrary units, indicating relative accumulation of anthocyanin pigments
