# Supplementary material for: Unlocking the Potential of Bacillus Strains for a Two-Front Attack on Wireworms and Fungal Pathogens in Oat
Source: Insects. 2025 Dec 24;17(1):28. doi: 10.3390/insects17010028 (PMC12841766; doi:10.3390/insects17010028)
Supplement: Supplementary file 1 [file insects-17-00028-s001.zip › insects-4006511-supplementary.pdf]

Table S1. DNA primers used for antibiotic and toxin gene presence screening and their expected product size (bp).

Gene	Primer	Sequence (5′ → 3′)	Product Size (bp)	Reference
srfAA	SRFA-F	TCGGGACAGGAAGACATCAT	201	Mora et al. (2011) [41]
	SRFA-R	CCACTCAAACGGATAATCCTGA		
bacA	BAC-F	CAGCTCATGGGAATGCTTTT	498	
	BAC-R	CTCGGTCCTGAAGGGACAAG		
fenD	FEND-F	GGCCCGTTCTCTAAATCCAT	269	
	FEND-R	GTCATGCTGACGAGAGCAAA		
bmyB	BMYB-F	GAATCCCGTTGTTCTCCAAA	370	
	BMYB-R	GCGGGTATTGAATGCTTGTT		
spaS	SPAS-F	GGTTTGTTGGATGGAGCTGT	375	
	SPAS-R	GCAAGGAGTCAGAGCAAGGT		
ituC	ITUC-F	GGCTGCTGCAGATGCTTTAT	423	
	ITUC-R	TCGCAGATAATCGCAGTGAG		
cry11	Un11-F	TTCCAACCCAACTTTCAAGC	305	Jain et al. (2017) [44]
	Un11-R	AGCTATGGCCTAAGGGGAAA		
cry1B	c1B-F	CAGAAACAACAGAACGACC	921	Thammasittirong and Attathom, (2008) [45]
	c1B-R	CACTTCCCCACCATCCAT		
vpb	Vip1-sc.fw	TATTAGATAAACAACAACAAGAATATCAATCTATTMGNTGGATHGG	585	Senthilkumar et al. (2021) [46]
	Vip1-sc.rev	GATCTATATCTCTAGCTGCTTTTTCATAATCTSARTANGGRTC		
vpa	Vip2-sc.fw	GATAAAGAAAAAGCAAAAGAATGGGRNAARRA	845	
	Vip2-sc.rev	CCACACCATCTATATACAGTAATATTTTCTGGDATNGG		
cyt1	cyt1gral-F	CCTCAATCAACAGCAGGGTTATT	477–480	Ibarra et al. (2003) [47]
	cyt1gral-R	TGCAAACAGGACATTGTATGTGTAATT		
cyt2	cyt2gral-F	ATTACAAATTGCAAATGGTATTCC	355–356	
	cyt2gral-R	TTTCAACATCCACAGTAATTTCAAATGC		

Table S2. Chemical analysis of the growing substrate used for pot experiments 1 and 2.

Basic chemical parameters									
pH KCl	pH H_2O	P_2O_5 (mg 100 g^{-1})	K_2O (mg 100 g^{-1})	N (%)	C (%)	SOM (%)	H_2O (%)		
4.57	5.33	11.95	28	17.4	40.7	77.03	31.23		
Content of microelements									
As (mg kg^{-1})	Cd (mg kg^{-1})	Co (mg kg^{-1})	Cr (mg kg^{-1})	Cu (mg kg^{-1})	Fe (mg kg^{-1})	Mn (mg kg^{-1})	Ni (mg kg^{-1})	Pb (mg kg^{-1})	Zn (mg kg^{-1})
7.45	BMDL	12.4	65.6	38.4	14,355	200	206	9.88	28.3
Content of macroelements									
Ca (g kg^{-1})	K (g kg^{-1})	K_2O (%)	Mg (g kg^{-1})	P (g kg^{-1})	P_2O_5 (%)				
9.63	1.21	0.15	4.08	1.21	0.28				

Table S3. Impact of *B. velezensis* BHC 3.1 and *B. thuringiensis* BHC 2.4 on nitrogen percentage in oat seeds and shoots.

	Treatment	Seed N%	Shoot N%
Pot experiment 1	Control	0.89 [c]	2.21 [b]
	Agriotes lineatus	0.86 [d]	2.05 [c]
	B. velezensis BHC 3.1 + *Agriotes lineatus*	1.17 [b]	2.47 [a]
	B. thuringiensis BHC 2.4 + *Agriotes lineatus*	1.46 [a]	2.35 [ab]
Pot experiment 2	Control	1.89 [bc]	2.36 [d]
	F. poae	1.77 [c]	2.13 [e]
	B. velezensis BHC 3.1	2.1 [ab]	3.28 [b]
	B. thuringiensis BHC 2.4	2.16 [a]	3.36 [a]
	B.velezensis BHC 3.1 + *F. poae*	1.9 [bc]	2.84 [c]
	B. thuringiensis BHC 2.4+ *F. poae*	2.01 [b]	2.81 [c]

Means followed by the same superscript letters are not significantly different according to Duncan's multiple range test ($p \leq 0.01$).
